# Hemorrhagic Shock as a Sequela of Splenic Rupture in a Patient with Infectious Mononucleosis: Focus on the Potential Role of Salicylates

**DOI:** 10.1155/2012/497820

**Published:** 2012-02-06

**Authors:** Konstantinos Bouliaris, Dimos Karangelis, Marios Daskalopoulos, Konstantinos Spanos, Michael Fanariotis, Anargyros Giaglaras

**Affiliations:** ^1^Department of General Surgery, General Hospital of Larissa, Tsakalof 1, 41221, Larissa, Thessaly, Greece; ^2^Department of Cardiovascular and Thoracic Surgery, University Hospital of Larissa, Mezourlo, 41334, Larissa, Thessaly, Greece; ^3^Department of Vascular Surgery, Attiko University Hospital, Rimini 1, 12462, Chaedari, Athens, Greece; ^4^Radiological Department, General Hospital of Larissa, Tsakalof 1, 41221, Larissa, Thessaly, Greece

## Abstract

Despite the fact that the vast majority of splenic ruptures are traumatic, infectious mononucleosis has been incriminated as a major predisposing factor that affects the integrity of the spleen, thus causing atraumatic ruptures and life-threatening hemorrhages. Herein we present a case of a 23-year-old Caucasian male who underwent an emergency laparotomy for acute abdomen and hemorrhagic shock, caused by spontaneous splenic rupture secondary to infectious mononucleosis. The potential role of salicylates in the development of a hemorrhagic complication in a patient with infectious mononucleosis is discussed.

## 1. Introduction

Infection with Epstein-Barr virus (EBV) has various clinical manifestations. Although in children, EBV infection is often asymptomatic, in adolescents and young adults, it typically manifests as infectious mononucleosis (IM) with fever, lymphadenopathy, and pharyngitis [1]. IM is a self-limited disease, in which pharyngitis resolves in approximately 7–10 days, fever in 7–14 days, and lymphadenopathy has a mean duration of 3 weeks. Splenomegaly is noted clinically in up to 50 percent of patients [1]. Although the course of the disease is usually benign, approximately 0.1–0.5 percent of patients with IM suffer spontaneous splenic rupture (SSR) [2]. In such cases with hemodynamic instability, splenectomy is the treatment of choice. On the other hand, hemodynamically stable patients who suffer from parenchymal spleen disruption of pathological substrate can be successfully managed nonoperatively using CT diagnosis, close clinical monitoring, and minimal transfusions [3]. Nonoperative therapy and splenic preservation in these patients can prevent postsplenectomy immunosuppression and infection, especially in the pediatric population, which is at higher risk for postsplenectomy sepsis [4].

Due to the resemblance of IM symptoms with those of common flu, patients tend to consume, usually without a physician's prescription, a salicylate medication. In the case presented herein, consumption of salicylates in high doses might have played a role in the final outcome.

## 2. Case Presentation

A 23-year-old male presented in the emergency department of a district general hospital with an acute onset of left upper quadrant abdominal pain and a syncopal episode one hour prior to his admittance. Abdominal pain had a sudden outburst while the patient was resting at home, and there was no indication of trauma history. The patient reported one-week history of fever up to 40°C, sore throat, and malaise, as well as oral consumption of 5 to 8 tablets of 500 mg of acetylsalicylic acid within the two previous days. On initial examination he was in shock, pale, tachycardic with a heart rate of 110 beats per minute, tachypnoeic and hypotensive with a blood pressure of 90/60 mm Hg, and a fever of 38.5 °C. Physical examination revealed bilateral nontender cervical lymphadenopathy and diffuse abdominal guarding with rebound tenderness. Kehr's sign was also positive. Admission laboratory tests revealed a WBC count of 17.000 cells/*μ*L with 53% atypical lymphocytes while the value of Hb was 9 g/dL. The patient's platelet count was normal (170.000/*μ*L), prothrombin time (PT) was 13.8 sec (reference time: 13 sec), international normalized ratio (INR) was 1.06, and activated partial thromboplastin (aPTT) time was 30.6 sec (normal: 28–40 sec). Serum transaminases were slightly elevated with an ALT 152 U/L and AST 138 U/L. The patient's history of fever, lymphadenopathy, sore throat, atypical lymphocytosis combined with his clinical presentation of peritonitis, and hypotension in the absence of a trauma incidence was suggestive of a possible diagnosis of spontaneous splenic rupture on the basis of the underlying IM.

**Figure 1:**
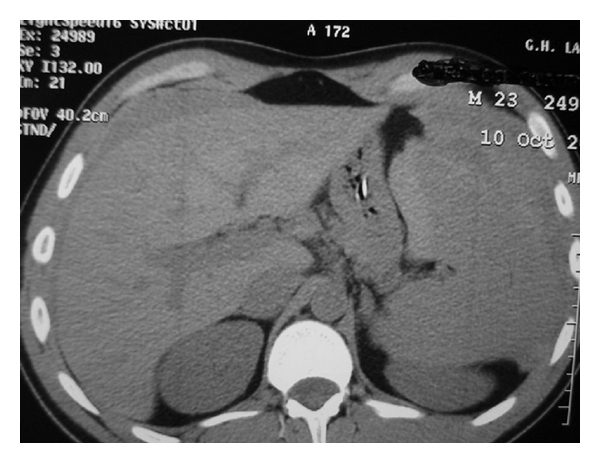
Preoperative CT scan indicative of the splenic rupture and hemoperitoneum.

 After initial resuscitation with 3 liters of intravenous crystalloids, an emergency abdominal CT was carried out to confirm the diagnosis. CT showed a large splenic hematoma and large amounts of fluid in all four abdominal quadrants and in the pelvis (Figure 1). An exploratory laparotomy was undertaken. During laparotomy, there was a notable amount of fresh and clotted blood in the abdomen while the spleen was large and congested. It had a 5 cm capsular tear near the hilum and a large subcapsular hematoma. The enlarged, friable spleen was almost completely devoid of capsule. We performed an uncomplicated splenectomy, and after a thorough lavage of the peritoneal cavity, no other abnormal findings were revealed. The patient returned to the surgical ward hemodynamically stable, and no blood transfusion was necessary. Pathologic examination revealed an enlarged spleen 15 × 7 × 5 cm, weighing 430 gr. Microscopic examination accented an expanded red pulp and moderate follicular hyperplasia. The diagnosis of IM was later confirmed by the presence of IgM antibodies to Epstein-Bar virus capsid antigen (VCA). The patient recovered uneventfully and was discharged on the 7th postoperative day.

## 3. Discussion

Spontaneous splenic rupture secondary to IM was first reported in the literature by King in 1941 [5]. Splenic rupture is a rare but potentially life-threatening complication of IM, estimated from 0.1% to 0.5% of clinically apparent IM [2]. It mostly occurs between the fourth and twenty-first days of symptomatic illness [6]. In our case, the spleen spontaneously ruptured 7 days after the onset of clinical symptoms of IM. According to the literature, Epstein-Barr virus is known to distort the splenic architecture by infiltrating the parenchyma with lymphocytes and atypical lymphoid cells [7]. This invasion compromises the fibrous support system of the spleen and thins out the splenic capsule, which predisposes to rupture [8]. Subsequently, the rupture occurs after minor trauma or spontaneously. The etiology behind spontaneous rupture is hypothesized to result from an acute increase in portal venous pressure caused by Vasalva maneuver, leading to vascular engorgement [9]. Sudden compression of the enlarged spleen secondary to contraction of the diaphragm or abdominal wall causes the thinned capsule to rupture [10].

Spontaneous splenic rupture has been associated with many other pathological conditions which compromise spleen's architecture [11]. Anticoagulants, fibrinolytics, and antiplatelet agents have also been cited as potential causes of SSR due to intrasplenic hematoma formation [12–14]. In the presented case, the subcapsular splenic hematoma formation might have been prompted by the large dose of acetylsalicylic acid the patient had consumed the last two days before the rupture. Salicylate drugs are often used as analgesics to relieve minor aches and pain, as antipyretics to reduce fever, and as anti-inflammatory medication. These drugs also have antiplatelet effects by inhibiting the production of thromboxane A_2 _ in platelets, producing an inhibitory effect on platelet aggregation. Salicylates are widely used and are easily available as over-the-counter medications; thus, they can be readily overused.

In our case, we hypothesize that the use of salicylates has possibly created a hemorrhaging substrate, which might had an impact on the already brittle spleen rupture due to the IM process. To our knowledge the possible association of the consumption of acetylsalicylic acid in IM-dependent splenic rupture has never been discussed before. In the vast majority of the previously reported cases of splenic ruptures in patients with IM the role of administered antipyretic drugs with antiplatelet effects has not been completely investigated. Although acetylsalicylic acid is a well-studied antiplatelet drug, its potential role in the pathogenesis of splenic rupture in patients with IM has not been explored.

## 4. Conclusions

The possibility for traumatic rupture of an enlarged spleen as a sequela of IM is well recognised and documented. Emergency splenectomy is a life-saving intervention in cases of splenic ruptures with hemodynamically instability. This report highlights the potential implication of acetylsalicylic acid in IM patients with splenomegaly. Although this case includes a clinical hypothesis, the authors strongly feel that all physicians must bear in mind this rare clinical scenario and remain vigilant should they encounter similar manifestations.
